# On the Reproductive Potential in *Primula veris* L. (Primulaceae): Embryological Features, Pollen and Seed Viability, Genetic Diversity

**DOI:** 10.3390/plants10112296

**Published:** 2021-10-26

**Authors:** Elina Yankova-Tsvetkova, Petka Yurukova-Grancharova, Ina Aneva, Petar Zhelev

**Affiliations:** 1 Institute of Biodiversity and Ecosystem Research, Bulgarian Academy of Sciences, 1113 Sofia, Bulgaria; y_grancharova@abv.bg (P.Y.-G.); ina.aneva@abv.bg (I.A.); 2 Department of Dendrology, University of Forestry, 1797 Sofia, Bulgaria; zhelev@ltu.bg

**Keywords:** embryology, pollen and seed viability, *Primula*, *Primula veris*, reproduction, reproductive potential

## Abstract

*Primula veris* (Primulaceae) is a valuable medicinal plant. The main characteristics for assessing the reproductive potential (embryological features; mode of reproduction; pollen and seed viability) and the genetic diversity of populations of the species from Bulgaria were studied. The anthers are tetrasporangiate. Their wall development follows the Dicotyledonous-type and consists of: epidermis, a fibrous endothecium, an ephemeral middle layer and a secretory (glandular) tapetum. After meiosis in pollen mother cells and simultaneous microsporogenesis tetrahedral tetrads are formed predominantly in the anthers. Many ovules (approximately 20) develop in the unilocular ovary and are anatropous, tenuinucellate and bitegmic. The embryo sac (ES) develops after *Polygonum* (monosporic)-type from the chalazal cell of linear megaspore tetrad in the ovule. After double fertilization, a Caryophyllad-type embryo and initially nuclear endosperm form. In the studied populations, high pollen viability of more than 95% was established. Extremely low viability (about 4%) of the seeds obtained from natural populations was established. The results reveal *P. veris* to be a predominantly amphimictic (sexually reproducing) species, although rare vegetative propagation is also observed. As a result of this study, essential data were obtained about the reproductive structures and processes and for assessing the reproductive potential of *P. veris*.

## 1. Introduction

The genus *Primula* L. includes six subgenera with 37 sections [1], and is the largest in the family Primulaceae. The number of species belonging to it, indicated by different authors, varies from about 400 up to more than 500 [1,2]. In Europe, the genus *Primula* is represented by only 34 species, included in four sections [1]. The genus was an object of the pioneer Darwin’s scientific treatise on the floral morphology, in particular heterostyly and reproductive biology [3]. He paid particular attention to the distyly in it. Heterostyly (reciprocal herkogamy) is confirmed for 28 Angiosperms families [4,5], and in particular, the distyly, as its most common type, is found in 26 of them [6], including Primulaceae.

*Primula veris* L. (cowslip, cowslip primrose) (syn. *Primula officinalis* Hill) belongs to the section *Primula*. The latter is phylogenetically quite isolated from the other sections in the genus and includes a total of seven species [7]. This section comprises six distylous and one homostylous species which all are diploids (2n  =  2x = 22) with basic chromosome number x = 11 [8,9]. In Bulgarian flora, the genus *Primula* is represented by eight species [10,11]. Specifically, the section *Primula* is represented by three species: *P. veris*, *P. elatior* and *P. vulgaris*.

The section *Primula* is used as a model for a broad area of studies: evolutionary [12], ecological and conservation [13], molecular and genetic [14,15], phytochemical and pharmacological [16,17], as well as studies related to hybridization [1,18], reproductive biology and pollination ecology [5,19], reproductive capacity [19], genetics, ecological basis and genetic control of the distyly [9,14,20], etc.

*P. veris* is a Euro-Siberian Temperate element [21] distributed throughout most of temperate Europe and Western Asia [22]. As with most species of the genus *Primula*, it is entomophilous with a great affinity between its flowers. Insect pollinators belong to Hymenoptera (mainly bumblebees and bees), *Lepidoptera* and *Diptera* [20,23].

*P. veris* has a long history of medicinal use. It is a species with an economic significance that is also used as a decorative and melliferous plant. In the European Red List of Medicinal Plants, *P. veris* is included under the category (LC) “Least Concern” [24]. Together with *P. elatior*, this species is listed in the European Pharmacopoeia as a source of *Primula* roots [25]. In Bulgaria, *P. veris* is included in the Biodiversity Act, Annex 4 [26], in particular in the list of species of medicinal plants under a special regime of protection and regulated use, as well as in the Medicinal Plants Act [27] in which *P. veris*, *P. elatior* and *P. vulgaris* are cited in the “List of Medicinal Plants Falling under the Provisions of this Act”. *P. veris* is a species collected as a herb with a commercial designation from its natural habitats, outside the territories of the Bulgarian national parks, determined by the Bulgarian Ministry of Environment and Water (MEW). The above-ground parts (exclusively flowers) and roots of *P. veris* are permitted to be harvested but only in accordance with the annual quotas of MEW.

Phenolic compounds, including flavonoids, phenolic acids, phenolic glycosides and triterpen saponins, are the main active compounds in *Primula* flowers and roots [28,29].

Many authors [17,30] have shown that the flowers and roots of *P. veris* and closely related species, specifically *P. elatior*, are used for the production of herbal teas and as dietary supplements. They exhibit various pharmacological activities, particularly secretolytic, expectorant, anti-inflammatory, diuretic, antimicrobial, antifungal, and sedative [28,29]. In official medicine, *P. veris* is used for treating bronchial catarrhs of the respiratory tract, pertussis, asthma, colds and influenza [17,31].

Contrary to the intensive studies undertaken on the members of the genus *Primula* in all aspects mentioned above, the embryological studies involve a small number of species. The data in them are usually scarce or incomplete.

The present study aimed to reveal the manner of reproduction, peculiarities of male and female generative spheres. The main parameters of reproductive biology (pollen and seed viability) support the reproductive success of Bulgarian populations of the valuable medicinal plant *P. veris*.

In the present work, Bulgarian populations of this species were studied for the first time with respect to embryological features, pollen and seed viability and genetic diversity. On the basis of the results, the possibilities of realization of *P. veris* reproductive potential can be used for its future successful introduction in culture. This is especially important, as its stocks as a valuable medicinal plant in Bulgaria are declining because the populations of *P. veris* are usually fragmented, small in size, and often subjected to anthropological or other adverse influences.

## 2. Results and Discussion

The distyly in genus *Primula* has become a textbook example of floral heteromorphy [32]. The studied species *P. veris* has a typical “distylous syndrome” [12,33]—a reproductive system maintaining high genetic variability which favors cross-pollination. In distyly, in the same population, there are plants with flowers with long styles and short stamens (i.e., long-styled or pin morph), and others with short styles and long stamens (i.e., short-styled or thrum morph) [34].

**Figure 1:**
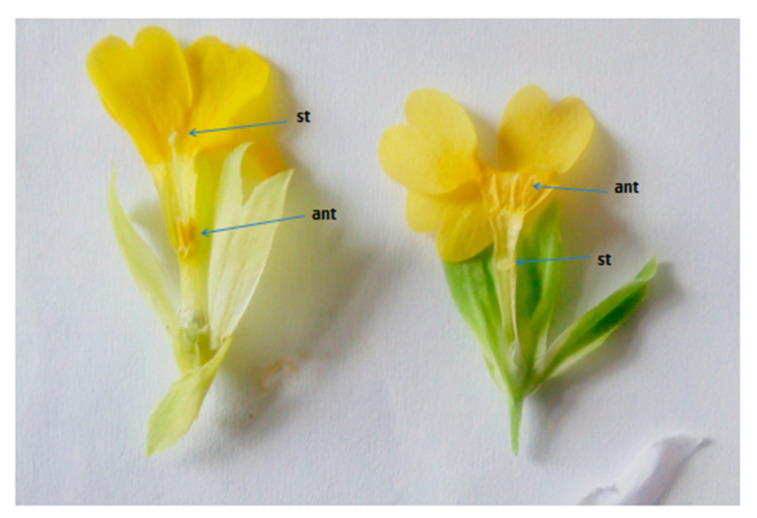
Distyly in *P. veris*: “pin” floral morph (to the left) and “thrum” flower morph (to the right), st—style; ant—anthers.

Our observation on the flower morphology of individuals of the two studied Bulgarian populations of *P. veris* show clear distyly, expressed in the presence of long-styled (pin morph) and short-styled (thrum morph) flowers illustrated in Figure 1. Here, it can be seen that in the former flower (pin morph) the anthers reach midway down the corolla tube, and in the latter one (thrum morph), they reach its mouth. In comparative terms is shown that the height of the short style found in the “thrum” morph corresponds to the position of anthers in “pin” morph [35]. Namely, the reciprocal position of male and female reproductive structures in the two-flower morph in *P. vulgaris* facilitates reciprocal pollination by insects [36] as in *P. veris*, both belonging to the section *Primula*.

It is important to note that in distyly, the long-styled floral morphs produce smaller in size but larger in amount pollen grains than the short-styled morphs. This was observed in *P. allionii* [37], *P. mistassinica* [38] and also in other distylous species [1,39].

### 2.1. Embryological Features

Some embryological features on species of the family Primulaceae, including *P. officinalis* (syn. *P. veris*), were reported by [40]. Since then, studies on the embryology of *P. veris* are almost lacking. Data for Bulgarian populations of the species were obtained for the first time in the present work.

#### 2.1.1. Anther, Microsporogenesis and Male Gametophyte Development

**Figure 2:**
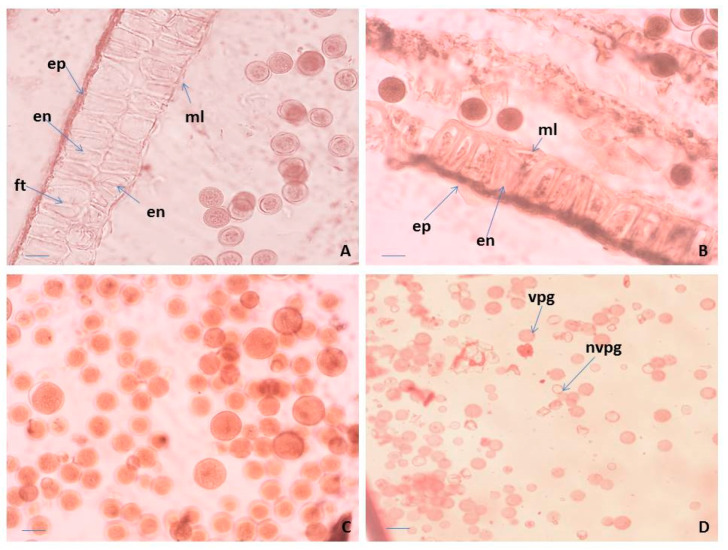
Anther and male gametophyte development. (**A**) Anther wall and mature pollen in an anther locule of “pin” floral morph, (**B**) anther wall and mature pollen, (**C**) mature pollen in an anther locule of “thrum” floral morph, and (**D**) viable and unviable pollen after acetocarmine testing. ep—epidermis; en—endothecium; ft—fibrous thickenings of the endothecium cells; ml—middle layer; vpg—viable mature pollen; nvpg—unviable mature pollen. Scale bar = 20 μm (**A**,**B**); 50 μm (**C**); 100 μm (**D**).

The anthers in *P. veris* are tetrasporangiate, a typical feature in all representatives of the family Primulaceae [41,42]. The anther wall forms following the Dicotyledonous-type [43] and consists of epidermis, endothecium, a middle layer and glandular (secretory) tapetum. Initially, it is difficult to distinguish morphologically the layers of the anther wall from each other. During the ontogenesis of the anther, they undergo significant changes and finally become clearly visible. The cells of the epidermis enlarge and lengthen tangentially, whereas these of the endothecium also extend but radially. In addition, formation of a second endothecium layer is observed, which has not been recorded so far in other *Primula* species. Thus, initially, the four-layered wall of the anthers in *P. veris* becomes five-layered. After forming microspore tetrads in the anthers, both endothecium layers develop fibrous thickenings (Figure 2A). During the anther ontogenesis, the cells of the middle layer elongate tangentially to such an extent that this layer becomes visible as a very thin strip between the endothecium and the tapetum. The tapetum cells in *P. veris* are one-nucleate. In contrast, in other Primulaceae members, namely in *Lysimachia hybrida* and *L. quadrifolia* [43], initially the one-nucleate tapetum cells become two-nucleate as a result of endomitosis, retaining their integrity. The glandular (secretory) anther tapetum, typical for the family Primulaceae [42], does not transform to an amoeboid one and remains cellular up to the end of the anther development.

Regarding the structure of the anther wall, in *P. bayernii* it is more massive than in *P. veris*, namely six-layered, consisting of epidermis, endothelium, two middle and two tapetum layers [44,45]. In the mature anthers of *P. veris*, some of the layers degenerate, and the wall contains only epidermis, endothecium with preserved integrity and remnants of the middle layer (Figure 2B). As a result of the normal course of meiosis in pollen mother cells (PMCs) and simultaneous microsporogenesis, predominantly tetrahedral microspore tetrads are formed in the anthers of *P. veris*. Mature pollen grains are bicellulate when released from the anther and morphologically identical or heterogeneous in size within the same anther locule (Figure 2A,C). In general, for almost all representatives of Primulaceae, it is stated that pollen grains are two-nucleate when dispersed [42,46].

Observations on the morphology of pollen from natural and cultivated Polish populations of *P. veris* show that pollen grains of the “thrum” (short-styled) morphs are more variable in size than those of “pin” (long-styled) morphs [47]. In the present study, this is illustrated in Figure 2A,C. In addition, it is important to note that in *P. veris*, a distylous species, the differences in the floral morphology do not influence the success of the insect pollination [20].

#### 2.1.2. Ovule, Megasporogenesis and Female Gametophyte Development

**Figure 3:**
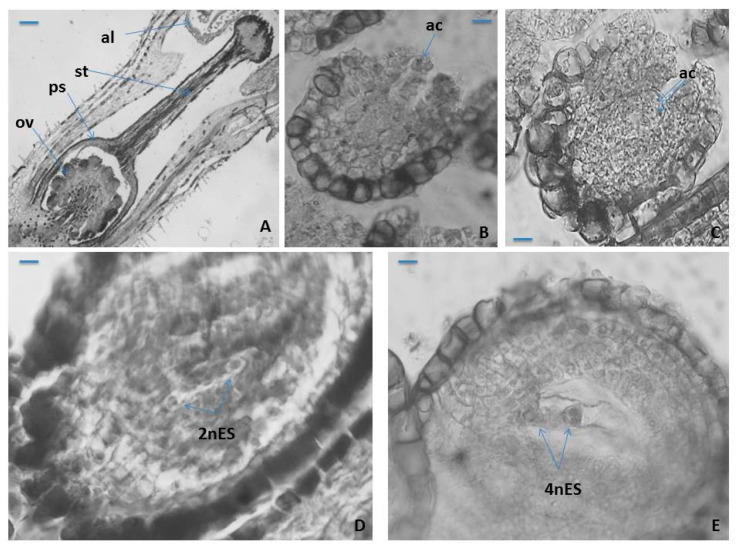
Ovule and female gametophyte development. (**A**) An ovary with ovules, style and stigma, (**B**) unicellular archesporium in a young ovule, (**C**) two-cellular archesporium in an ovule, (**D**) two-nucleate *Polygonum*-type ES and (**E**) Four-nucleate *Polygonum*-type ES. ov—ovule; ps—ovary; st—style; al—anther locule; ac—archesporium cell; mt—megaspore tetrad; 2nES—two-nucleate ES; 4nES—four-nucleate ES. Scale bar = 100 μm (**A**); 50 μm (**B**,**C**); 20 μm (**D**,**E**).

The ovary in *P. veris* is superior, unilocular, in which approximately 20 ovules with free central placentation being formed and developed (Figure 3A). In *P. vulgaris* [48] and *P. nutans* [49], the ovule number in an ovary does not differ (it is almost the same) between “pin” and “thrum” floral morphs. In the pistil of *P. veris* a solitary style and papilous capitate stigma (with) were observed (Figure 3A). The developed ovules in *P. veris* are anatropous, tenuinucellate, bitegmic with two-layered outer integument and more massive (four- to five-layered) inner integument (Figure 3A). The same type of ovule was reported for most Primulaceae representatives [41,42,46] and in particular for *P. officinalis* and *Cortusa matthioli* [40]. It has been established that the ovules in *P. amoena* are not anatropous but hemianatropous [50]. In another member of Primulaceae, namely *Anagallis pumila*, a gradual change to apotropous and even campylotropous ovule types have been observed [51].

The cells of the epidermal layer of the outer integument in *P. veris* and of the endothelium have strongly thickened walls. During the ovule ontogenesis and the formation of the embryo sac (ES) in it, the cells of the innermost layer of the inner integument lengthen radially becoming tabular in form, forming so-called endothelium (integumentary tapetum) [52], which after the degeneration of nucellus surrounds the ES (Figure 3B). Hypodermally, in the nucellus of an ovule, unicellular archesporium forms (Figure 2B), but rarely, two archesporium cells are also observed (Figure 3C). The formation of two archesporium cells, instead of one, has also been occasionally found in the ovules of another Primulaceae member, namely *Anagallis pumila* [51]. Anderberg’s statement [46] that the multicellular female archaespore is typical of the whole family Primulaceae raises doubts since in the embryological studies conducted so far, such feature has not been found, including in *P. veris*.

Contrary to Anderberg’s assertion, in important treatises on Angiosperm embryology, for the family Primulaceae, the formation of only one archaesporium cell in the ovule is indicated [41,42].

In *P. veris*, as in other *Primula* species, namely *P. bayernii* [44], *P. algida* and *P. amoena* [50], the archesporium cell functions directly as megaspore mother cell (MMC), and because of that, no cover cell formation is observed. After the meiosis occurring in MMC, a linear megaspore tetrad forms in the ovule and from its chalazal megaspore *Polygonum* (monosporic)-type ES (Figure 3D,E) develops, which is typical for the family Primulaceae [41,42]. Except for the linear megaspore tetrads, formation of T-shaped tetrads in some ovules of *Anagallis pumila* has also been observed [51].

**Figure 4:**
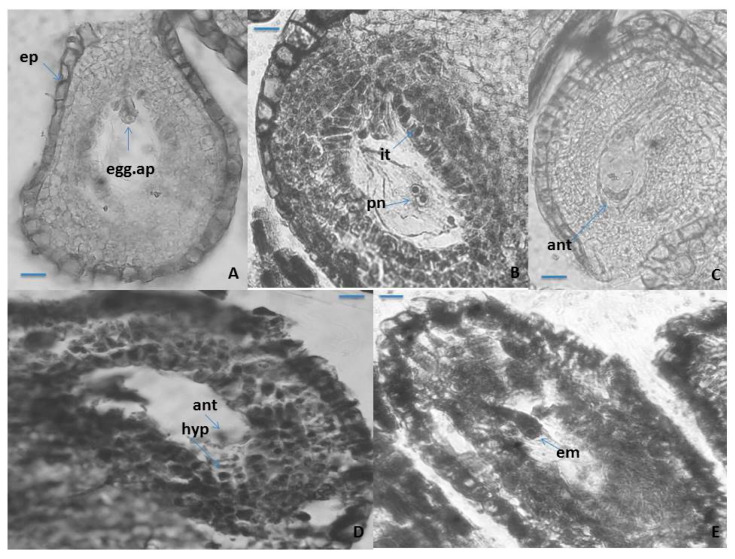
Ovule and development of the female gametophyte. (**A**–**C**) Anatropous ovule with mature *Polygonum*-type ES, (**D**) antipodals and hypostase in the ES cavity, and (**E**) young globular embryo with suspensor in the ES cavity. ep—epidermis; egg.ap—egg apparatus; it-integumentary tapetum pn—polar nuclei; ant—antipodals; hyp—hypostase; em—embryo. Scale bar = 20 μm (**A**–**E**).

The mature seven-cellular *Polygonum*-type ES consists of the following elements: an egg apparatus, with small size of its three cells (egg cell and two synergids), with the typical shape and location of the nuclei in them (Figure 4A); central cell of the ES, results of the fusion of the two polar nuclei about the time of fertilization (Figure 4B), and three ephemeral antipodals, that degenerate before the fertilization (Figure 4C). The ephemerous nature of antipodals is shown as a typical feature for the whole family Primulaceae [41,42,45,46]. The spherical inclusions in the epidermal cells of the anther wall and in the epidermal cells of the outer integument of the ovule (Figure 2B and Figure 4A), observed in some other representatives of Primulaceae, are probably tannins [45]. It was observed in *P. veris* that under the antipodals, from cells of the internal integument an additional structure in the chalaza is formed, namely a hypostase (Figure 4D). This is a newly established feature that has not been reported so far for the embryology of the genus *Primula* or of the family Primulaceae. The embryo and endosperm formations begin after fertilization of the egg cell and the central cell of the ES, respectively, and the endospermogenesis precedes embryogenesis. This was found in all studied members of the family, and differences were found only between the time of the first division of the zygote and the number of the already formed free endosperm nuclei. The embryogenesis in *P. veris* runs after the Caryophyllad-type (Figure 4E), which is typical for the family Primulaceae [41,42,46], and the embryo has a long suspensor at its globular stage of development (Figure 4E). Initially, the endosperm consists of free nuclei, but during embryogenesis, it transforms into a cellular one. It has been observed that cytokinesis in *P. veris* and other embryologically studied *Primula* species occurs only after different number of free endosperm nuclei have been formed, for example, 32–46 endosperm nuclei in *P. algida* and 64–128 in *P. amoena* [50], after the stage of the globular embryo in *P. bayernii*, and in *P. veris* the presence of a thousand endosperm nuclei has been found during the first division of the zygote [40]. In *P. amoena*, the formation of endosperm haustorium has been after the endosperm is already in its cellular stage of development, as well as several lateral haustoria penetrating to the cell of the endothelium [50]. The presence of endosperm haustorium has not been reported in the general embryological characteristics of the family Primulaceae [41,42,46].

**Figure 5:**
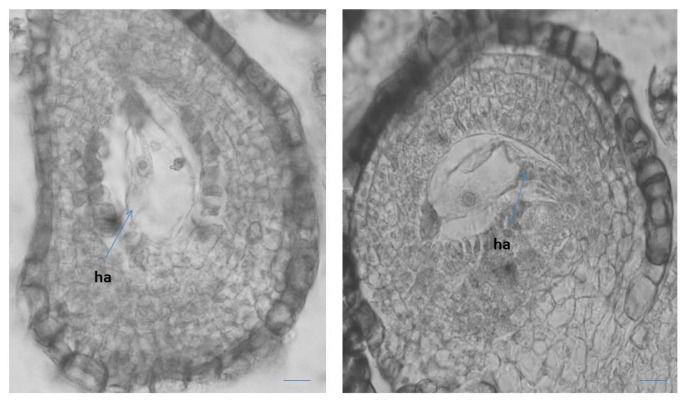
Central cell in the ES with outgrowth like haustorization (ha) to the chalaza of the ovule. Scale bar = 20 μm.

It has been observed that after the degeneration of the antipodals (before fertilization), the lower end of the central cell enlarges considerably, forming an outgrowth with dense cytoplasm haustorizing to the chalaza (Figure 5). Similar haustorization of the central cell of the ES was also observed in two other *Primula* species—*P. algida* and *P. amoena* [50]. In *Primula amoena*, after the endosperm has transformed from nuclear to cellular, the formation of endosperm haustorium is observed, as well as several lateral haustoria penetrating into the endothelial cells [50]. The formation of such structures was not detected in the present study of *P. veris*. In addition, the formation of endosperm haustorium is not included as a characteristic feature of the Primulaceae family in well-known monographs on the embryology of flowering plants [41,42,46].

This study shows that *P. veris* is a diploid, sexually reproducing (amphimictic) species that reproduces mainly by seeds. However, rarely it may reproduce vegetatively during the growing season by the formation of lateral rosettes. This has already been announced for the species [33], and is confirmed by our observations (in situ and ex situ). Results of a study on the distyly and its influence on the size of the Belgian populations of *P. veris* [53] show that in large populations, the short-styled morph is usually more “female based”, and the long-styled morph may rather manifest as a pollen donor. The same authors [53] also point out that these two morphs may behave differently with respect to restrictions arising in fragmented populations. In Bulgarian flora, *P. veris* is represented by such populations.

It is important to note that in our study of the generative sphere of *P. veris*, neither apomixis nor formation of additional embryos (i.e., polyembryony) was observed. As a result, the species is considered sexually reproducing, which correlates with its diploid status. However, polyembryony is found in other *Primula* species. Therefore, in *P. auricula* the additional 2–3 globular embryos are formed by the zygote [54] and this type of polyembryony is described as “cleavage polyembryony” [55]. In *P. amoena*, besides the zygotic embryo from the fertilized egg cell, an apomictic embryo also forms [50] from the synergid, without fertilization (apogamy).

### 2.2. Pollen Viability

Pollen viability (fertility) is an important factor in whether a population will undergo effective pollination and subsequent sexual reproduction to ensure the survival of each plant species. Different terms in pollen testing criteria based on the stages of pollen development in which it is tested are suggested [56]. In our study, we use the term “viability”, which is defined as “having the capacity to live, grow, germinate or develop”, which refers to the assessment of the viability of mature pollen [57].

According to the level of staining, the pollen viability in *P. veris* was estimated after acetocarmine testing: red-colored pollen grains were determined as viable, and colorless ones as nonviable (Figure 2D). As a result, the following percentage of pollen viability was found: 98.05% ± 2.2 for the Pirin Mts (the Ilindentsi village) population and 95.84% ± 5.7 for the population from the Golo Bardo Mts.

The pollen viability is one of the main parameters for the evaluation of plant reproductive potential. The viable (fertile) mature pollen is that which, when falling on the stigma under normal conditions, would start growing a pollen tube and finally discharge its male gametes in the embryo sac in an ovule to perform fertilization. A comparative study on “pin” and “thrum” floral morphs in *P. paliurni* showed that the pollen viability is significantly affected by the temperature and humidity [58]. In the plants from a population of this species, these authors [58] found that the pollen of the “thrum” morph showed significantly higher viability than the pollen of the “pin” floral morph. While in *P. bayernii*, pollen sterility in the “pin” flowers was found [44], the data from our study in *P. veris* shows that in both “pin” and “thrum” flowers, fertile pollen predominantly forms. The latter is an important factor for the efficiency of the subsequent processes of pollination and fertilization, and hence, the sexual reproduction of this species.

### 2.3. Seed Viability

A viable seed is a seed that is capable of germinating and producing a “normal” seedling [59]. A related term is ‘seed viability’, which describes the share of viable seeds and is usually used synonymously with ‘germination capacity’ (germinability). This definition includes dormant but viable seeds, in which case the dormancy must be broken before viability can be assessed if germination is achieved. Therefore, a non-viable seed fails to germinate even under optimal conditions, including treatments for the removal of dormancy. Seed germination is “the emergence and development from the seed embryo, those essential structures, which, for the kind of seed in question, are indicative of the ability to produce a normal plant under favourable conditions” [60].

**Figure 6:**
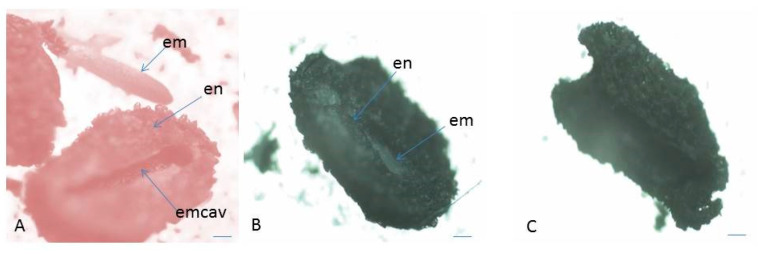
Assessment of seed (embryo) viability in *P. veris* after TZ testing. (**A**) Seed with endosperm and viable embryo, (**B**) seed with endosperm and unviable embryo, (**C**) empty seed (without embryo) unnviable. em—embryo; en—endosperm; emcav—ES cavity. Scale bar = 100 μm.

**Figure 7:**
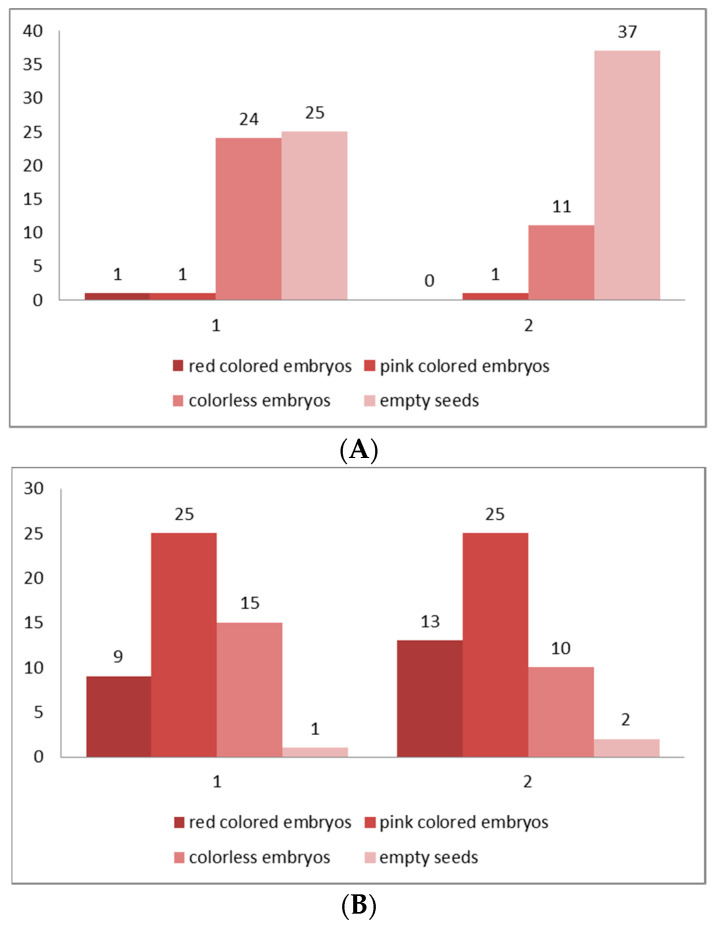
(**A**,**B**) Classification of the embryos viability in four classes according to the intensity of their staining after the results of TZ testing. (**A**) From seeds collected from the natural populations (in situ), (**B**) from seeds collected from the plants at the experimental plots (ex situ). Class I—seeds with viable embryo; completely (100%) colored in dark red; Class II—seeds with viable embryo; colored in pink; Class III—seeds with colorless embryo, unviable; Class IV—empty seeds without embryo. 1—*Golo Bardo* Mt population; 2—the *Ilindentsi village* population.

In the present study, the assessment of seed (embryo) viability was made after testing with tetrazolium solution in the two studied populations (both in situ and ex situ collected seeds) of *P. veris* (Table 1 and Table 2). The evaluation was performed on the basis of the criteria for interpretation of the tetrazolium test results, according to the intensity of staining and localization of unstained parts in the embryo [61]: the embryos stained entirely in dark red, brighter red or pink and partially stained embryos (only the root tip colored in red) are defined as viable; colorless or partially stained embryos (only the top of the embryo–cotyledons are colored in red), as well as empty seeds are considered non-viable (Figure 6 and Figure 7). The color differences observed, together with the knowledge of seed features and function, permit an assessment of the presence, location, and nature of weaknesses within embryo tissues [61].

In distylous species such as *P. veris*, only cross-pollination between the two genetically determined individuals (“pin” and “thrum” morphs) produces a successful seed set [62]. The established exclusively low seed viability for the natural populations (in situ)—4% for Golo Bardo Mt population and 2% for the Ilindentsi village population (Table 1)—is probably due to the seed dormancy, and to various restrictions in the conditions in the natural habitats, which negatively influence the processes of reaching seeds maturity and their further development in plants. The results of studies on foreign populations of *P. veris* have also shown an exclusively low (0.01%) seed viability [19,63]. The higher seed viability (over 60%) (Table 2) established in ex situ cultivated plants from the two studied populations shows a positive influence of the controlled conditions on the formation and maturation of *P. veris*. It is a prerequisite for the successful growth of the species in culture.

The exclusively low seed viability assumes a slow rate of germination. It decreases the successful growth of the seedling progeny in *P. veris*, and one of the probable reasons for this may be seed dormancy [64]. It has been shown that the low seed viability, which is a reason for the poor germination rate, is due to dormancy [65]. The seed dormancy in *P. veris* continues during the last several months of its development [64,66]. The non-mature seeds are strictly dormant at the time of dispersal and to activate germination a cold stratification is required [67].

### 2.4. Genetic Diversity

In total, 63% of the primers scored for the initial pilot study of *Primula veris* were polymorphic. The mean number of bands per primer was 10.7. These data are similar to those obtained by Crema et al. [68] for the endemic species *Primula apennina*, but there the authors used many more primers (twenty-five primers).

The percentage of polymorphic bands was the highest in the population of Western Rhodopes (74), which is due probably to the larger number of the studied individuals. The same percentage in the population of Petrohan was 72, in the population of Slavyanka 69, and in the population of Gorno Harsovo (Rila) 67. The number of polymorphic bands is only approximately characteristic of the genetic diversity, and is analogous to the mean number of alleles in other markers (for example, isozymes). Much more informative are the characteristics of genetic diversity [69]. When using this index, again the highest diversity was demonstrated by the population of Kraishte (Western Rhodopes) (0.256), followed by Slavyanka (0.238), Gorno Harsovo (0.201), and the lowest level of diversity was recorded in the population of Petrohan, which is the northernmost of all of the populations.

The Shannon’s polymorphic index showed similar trends and again the highest values had the population from the Western Rhodopes, followed by Petrohan, Gorno Harsovo, and the lowest value was recorded in the population of Slavyanka.

**Figure 8:**
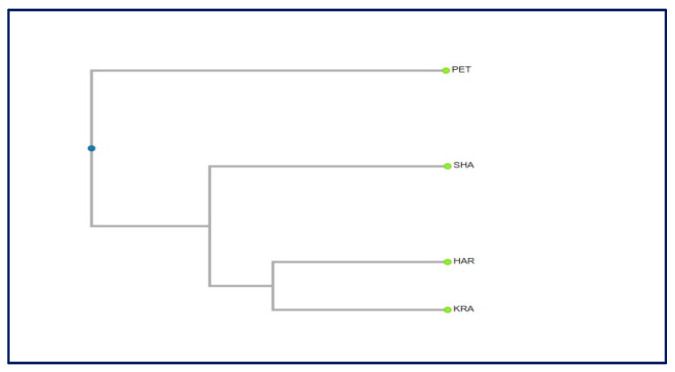
Dendrogram based on the genetic distances among the studied populations.

The dendrogram constructed based on the genetic distances among populations revealed that there is no relationship between the geographic and genetic distances (Figure 8). The population Petrohan (Western Stara planina) can be clearly distinguished, and the other three populations are closer to each other. The lack of clear spatial subdivision in the grouping of populations is probably due to the small sample size. It would be worth including other populations in the study, and then we could expect to get more clear relationships and trends in the distribution of the genetic diversity and to draw more definite conclusions.

As in *P. apennina* [68] and *P. heterochroma* [70], the level of genetic variability in *Primula veris* was high both at species and at population levels (Table 3). Therefore, it can be concluded that the high genetic diversity is common for *Primula* species. It correlates with their outcrossing nature, which is a prerequisite for high level of genetic diversity within populations and lower differentiation among populations [71].

## 3. Materials and Methods

### 3.1. Study of Reproductive Capacity

The study of embryological characteristics, pollen and seed viability was carried out on two natural populations of *Primula veris* distributed in Bulgaria: 1. Pirin Mts, in a pine forest above the village of Ilindentsi; 2. Znepole region, Golo Bardo Mt over the town of Pernik. Voucher specimens are deposited in the Herbarium of the Institute of Biodiversity and Ecosystem Research (SOM), Sofia. In Bulgaria, this species is widespread in the plains, foothills, and mountains, from sea level to above 2500 m altitude [10]. It is often represented by populations that are small in size and usually fragmented. Karyological studies on Bulgarian populations reveal that *P. veris* is a diploid species with chromosome number 2n = 22. This chromosome number is also shown for populations from the Znepole Region [72] and Pirin Mts [73].

#### 3.1.1. Embryological Study

The material used for the embryological study is collected from the two native populations (above mentioned), as well as from plants transferred and grown in greenhouse conditions at the Institute of Biodiversity and Ecosystem Research, Bulgarian Academy of Sciences, Sofia (Table 1). The voucher specimens were deposited in the Herbarium of the Institute of Biodiversity and Ecosystem Research (SOM), Sofia. For the embryological study, flower buds, open flowers and seeds at different stages of their development were collected during May–July and fixed in the FAA mixture (formalin: glacial acetic acid: 70% ethanol in correlation 5: 5: 90 parts). Then, the fixed material was treated according to the classical paraffin methods [74]. The serial paraffin sections were made with rotary microtome “Leitz” with 6–15 µm thickness (depending on the age of the material) and stained with Heidenhain’s iron haematoxylin [75]. The prepared permanent slides were mounted in Entellan. The observations on permanent slides were made with a light microscope “Olympus” CX21. The microphotographs were taken with a digital camera “Infinity lite”, 1,4 Mpx.

#### 3.1.2. Pollen Viability

The estimation of pollen viability of the two studied populations of *P. veris* was made after using the acetocarmine test [76]. For this purpose, 30 mature anthers isolated from fully open flowers from plants of each of the two populations studied were crushed on the microscopic glass slides and the pollen grains released from them were treated in a drop of 1% acetocarmine solution and as a result, the cytoplasm of viable pollen stains red. At the same time, it remains unstained, almost transparent in unviable pollen.

Assessment of the pollen viability was performed by direct counting, using the light microscope “Olympus” CX21 (on the visible field at magnification 100× and 400×, depending on their size).

#### 3.1.3. Seed Viability

To assess seed viability (fertility), the tetrazolium test [77] was applied on 50 mature seeds from plants of each of the two studied populations from the natural habitats (in situ) and from the experimental plots (ex situ) of the Institute of Biodiversity and Ecosystem Research, under controlled conditions (Table 1 and Table 2, respectively). First, the mature seeds were pre-soaked in water at 30–35 °C and then were pricked and incubated with 1% solution of 2, 3, 5-triphenyl tetrazolium chloride for 24 h at 25 °C. The principle of TZ testing is based on dehydrogenase activity in the viable seed tissues during the respiration process when dehydrogenase catalyzes initially colorless 2,3,5 triphenyl tetrazolium chloride solution into red stained formazan. If the embryo goes pink or red, there are living tissues with high respiratory activity, which has reduced the tetrazolium chloride (colorless) to formazan (pinkish-red) via dehydrogenase enzyme. The seeds (embryos) with lower viability and colorless embryos were considered unviable (unfertile). Thus, assessing the staining intensity, the seeds (embryos) were evaluated (Figure 1). The observations and microphotographs for assessment of the seed viability were made with light microscope “Olympus” CX21 and an “Infinity lite” digital camera 1.4 Mpx, respectively.

### 3.2. Study of Genetic Diversity in the Target Species

The genetic study was performed in four natural populations distributed throughout the whole range of the species’ distribution in Bulgaria (Table 3): Petrohan (Western Stara planina), Gorno Harsovo (Rila Mts), Kraishte (Western Rhodopes), Shabran (Slavyanka Mts). Leaves from ten randomly chosen individuals per population were sampled for the analysis.

DNA was extracted by Invisorb Spin Plant Mini Kit (Invitek Molecular GmbH, Berlin, Germany), following the protocol of the producer. DNA quantity and quality before the analysis were measured by the spectrophotometer Nanodrop Lite (Thermo Fisher Scientific).

Six primers for obtaining Internal simple sequence repeats (ISSR) genetic markers were applied in the study (Table 4). The markers were successfully applied to other *Primula* species [68]. The amplification reaction and electrophoresis followed the method described by [78].

Binary matrix was constructed using presence (1) and absence (0) of particular band. Based on the matrix, the following parameters were calculated: expected heterozygosity and the percent of polymorphic bands. Nei’s genetic distances among the population pairs were calculated for revealing the inter-population variation. Cluster analysis was applied by using UPGMA method and by means of the software ClustVis [79].

## 4. Conclusions

In this study, for the first time, the peculiarities of the generative processes and structures of the Bulgarian populations of *Primula veris* are established, in order to reveal the reproductive potential of this valuable medicinal plant. It should be noted that some new important reproductive characteristics have been identified in this species, as follows: development of two fibrous layers in the anther wall instead of one and formation of hypostase as an additional structure in the chalasal part of the ovules. Considering that the embryological features are very conservative, the established new features enrich the data on embryology and reproductive biology of this species and the genus *Primula*. The study revealed *P. veris* is exclusively sexually reproducing in the Bulgarian populations. No apomixis or at least some of its elements is found which correlates with the diploid status of the species.

The normal (without deviations) course of the processes of formation of the male and female gametophyte, with relatively few observed degenerations of ovules and embryo sacs, combined with the formation of large quantity of fertile pollen (over 95%), provide high reproductive potential and successful reproduction of the species, especially under appropriate conditions. The specificity of *P. veris* is exclusively determined by the fact that it is a distylous, and therefore a self-incompatible, species. Therefore, it is sensitive to pollen limitation [80], and low habitat quality may affect pollinator densities negatively [19]. Consequently, the small populations are at increased long-term risk of extinction, because they are less able to respond to changes in the environment [81], and in particular, this is likely to be true for the studied fragmented populations of *P. veris*, widespread in Bulgaria.

The study on genetic diversity revealed that *P. veris* exhibits similar patterns of variation to those of closely related species with similar life-history characteristics. The revealed high genetic variability in the studied populations corresponds to that established in other *Primula* species and allows the conclusion that the high genetic diversity is a common feature of *Primula* species, due to their outcrossing mode of reproduction. However, a more complete picture can be obtained by studying more populations from the whole species range.

The results on the reproductive potential show that the species can be successfully cultivated in ex situ conditions to obtain medicinal drugs. This option is especially valuable, bearing in mind that the species, although widespread throughout the whole country, is represented by populations that are small in size and fragmented.

Therefore, we consider that further thorough investigations on morph-specific reproductive processes in *Primula veris* must be carried out in a context of preservation of the size and character of its natural populations.

## Figures and Tables

**Table 1:** Assessment of the seed (embryo) viability after TZ testing for seeds collected from the natural populations (in situ).

Population	Number of Tested Seeds	Number of Viable Embryos	Viable Embryos %	Unviable Embryos %
Golo Bardo Mt	50	2	4	96
Ilindentsi village	50	1	2	98

**Table 2:** Assessment of the seeds (embryos) viability after TZ testing for seeds collected from the plants at the experimental plots (ex situ).

Population	Number of Tested Seeds	Number of Viable Embryos	Viable Embryos %	Unviable Embryos %
Golo Bardo Mt	50	34	68	32
Ilindentsi village	50	38	76	24

**Table 3:** Natural populations included in the genetic studies and polymorphism and diversity.

Population	Geographic Coordinates	Altitude (m)	Percent of Polymorphic Bands	Gene Diversity He (±SD)	Average Intra-Population Diversity H_S_ (±SD)
Shabran (Slavyanka Mts)	41°23′19″ N 23°36′30″ E	1900	69	0.238 ± 0.155	0.236 ± 0.249
Kraishte (Western Rhodopes)	41°57′28″ N 23°39′12″ E	1180	74	0.256 ± 0.108	0.302 ± 0.248
Petrokhan (Western Stara planina)	43°7′39″ N 23°7′28″ E	1450	72	0.192 ± 0.134	0.256 ± 0.154
Gorno Harsovo (Rila Mts)	42°00′47″ N 23°12′30″ E	820	67	0.201 ± 0.164	0.238 ± 0.203

**Table 4:** ISSR primers studied for the study of genetic diversity of *Primula veris*.

ISSR Primer (Name and Sequence)	Optimal Annealing Temperature	Total Number of Bands	Number of Polymorphic Bands
UBC-807 AGAGAGAGAGAGAGAGT	59	16	11
UBC-808 AGAGAGAGAGAGAGAGC	57	10	8
UBC-811 GAGAGAGAGAGAGAGAC	53	12	10
UBC-827 ACACACACACACACACG	57	6	5
UBC-835 AGAGAGAGAGAGAGAGYC	54	19	17
UBC-845 CTCTCTCT CTCTCTCTRG	54	21	16

## Data Availability

Not applicable.
